# Effectiveness of automatic tube potential selection with tube current modulation in coronary CT angiography for obese patients: Comparison with a body mass index-based protocol using the propensity score matching method

**DOI:** 10.1371/journal.pone.0190584

**Published:** 2018-01-05

**Authors:** Hong Seon Lee, Young Joo Suh, Kyunghwa Han, Jin Young Kim, Suyon Chang, Dong Jin Im, Yoo Jin Hong, Hye-Jeong Lee, Jin Hur, Young Jin Kim, Byoung Wook Choi

**Affiliations:** Department of Radiology, Research Institute of Radiological Science, Severance Hospital, Yonsei University College of Medicine, Korea; Faculty of Medical Science - State University of Campinas, BRAZIL

## Abstract

**Background:**

Reduced image quality from increased X-ray scatter and image noise can be problematic when coronary computed tomography angiography (CCTA) imaging is performed in obese patients. The aim of this study was to compare the image quality and radiation dose obtained using automatic tube potential selection with tube current modulation (APSCM) with those obtained using a body mass index (BMI)-based protocol for CCTA in obese patients.

**Methods:**

A total of 203 consecutive obese (BMI > 30 kg/m^2^) patients were retrospectively enrolled, of whom 96 underwent CCTA with APSCM and 107 underwent a BMI-based protocol. After applying the propensity score matching method, the clinical parameters, subjective and objective image quality, and radiation dose were compared between the APSCM group and the matched BMI-based group. These parameters were also compared among different tube potential subgroups.

**Results:**

No significant differences were observed between the APSCM group and the BMI-based group with respect to image quality or radiation dose assessment (*p* > 0.05). Twenty patients (21%) examined with 140 kV in the APSCM group were exposed to significantly more radiation (*p* < 0.05) than patients in the BMI-based group or patients in the other APSCM kV subgroups; significant improvement in image quality was not observed in the 140 kV subgroup. Patients with a high BMI and a large effective diameter tended to be examined with 140 kV (*p* < 0.05).

**Conclusion:**

The use of APSCM for CCTA in obese patients did not significantly reduce the radiation dose or improve image quality compared with those in the matched BMI-based group. Our data indicate that it is better to avoid using APSCM when 140 kV is automatically selected, due to increased radiation dose and lack of significant improvement in image quality.

## Introduction

The prevalence of obesity has been substantially increasing and has become a major health problem worldwide [1]. Despite the high risk and mortality rate of coronary artery disease (CAD) in obese patients, most anatomical imaging techniques for evaluation of CAD—including coronary computed tomography angiography (CCTA)—pose special challenges when used with obese patients [2]. Reduced image quality from increased X-ray scatter and image noise can be problematic when CCTA imaging is performed in obese patients, hampering delineation of small vessels and noncalcified plaques [3, 4].

Automatic tube potential selection with tube current modulation (APSCM) has recently been applied to computed tomography (CT) imaging. The APSCM software program automatically selects the appropriate tube potential and modulates the tube current-product settings for each patient based on body size, attenuation profiles obtained from topograms, and the user-chosen contrast-to-noise ratio (CNR). Due to the more frequent use of low tube potentials (80 kV or 100 kV), the application of APSCM to CCTA reduces the radiation dose compared with the body-mass index (BMI)-based protocol, while still maintaining subjective image quality [5–11].

However, obese patients are more frequently examined with high tube potentials (i.e., 140 kV) when APSCM is used than when BMI-based protocols are used [11]. While these high potentials may reduce image noise, the higher contrast enhancement seen with low tube potentials may be lost. Therefore, it is unclear whether there is an overall benefit to obese patients in terms of radiation dose and image quality when APSCM is used. We hypothesized that the use of APSCM in obese patients would result in increased radiation doses without obvious improvement in image quality compared with the conventional BMI-based protocol, due to the frequent use of high tube potentials.

The purpose of this study was to compare the image quality and radiation dose obtained using APSCM with those obtained using a BMI-based protocol for CCTA in obese patients.

## Materials and methods

### Patient population

**Fig 1:**
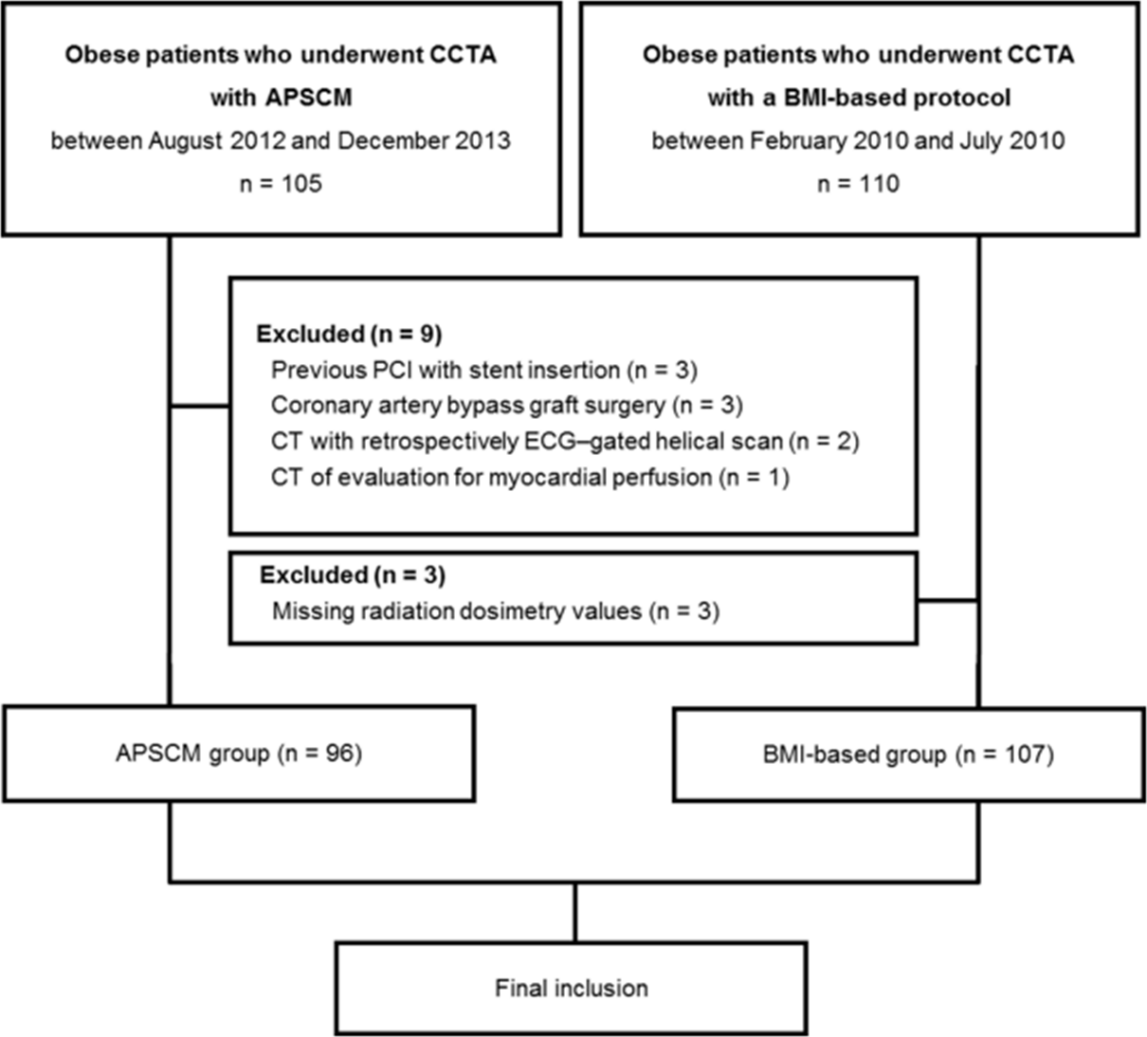
Flow chart of study population. CCTA, coronary computed tomography angiography; APSCM, automatic tube potential selection with tube current modulation; BMI, body mass index; PCI, percutaneous coronary intervention; ECG, electrocardiography.

This study was approved by the Severance Hospital Institutional Review Board, and the requirement for informed consent was waived. A total of 105 obese patients (BMI > 30 kg/m^2^) who underwent clinically indicated CCTA using APSCM from August 2012 to December 2013 were consecutively enrolled in this study. The control group consisted of 110 consecutive obese patients who underwent CCTA from February to July 2010, according to our institution’s previous BMI-based examination protocol (BMI-based group). In the APSCM group, nine patients were excluded because they underwent percutaneous coronary intervention with stent insertion (n = 3), coronary artery bypass graft surgery (n = 3), CT with a retrospective electrocardiographically (ECG)-gated helical scan (n = 2), or CT for evaluation of myocardial perfusion (n = 1). In the BMI-based group, three patients were excluded because of missing radiation dosimetric values. The final study population consisted of 96 patients in the APSCM group and 107 patients in the BMI-based group (Fig 1).

### CT acquisition and reconstruction protocol

All patients underwent CCTA with a second-generation dual-source CT scanner (SOMATOM Definition Flash; Siemens Healthcare, Forchheim, Germany). In the absence of contraindications, patients with a heart rate (HR) greater than 65 beats per minute received a 50-mg dose of oral b-blocker (metoprolol tartrate) 1 hour before the examination and a 0.3 mg sublingual dose of nitroglycerin just before scanning. The data acquisition mode was determined according to the HR in both groups. For patients with a regular HR < 60 beats/min, scans were performed in the prospectively ECG-triggered high-pitch spiral mode (FLASH mode). For patients with a regular HR ≥ 60 beats/min, scans were performed in the prospectively ECG-triggered axial mode (axial mode). If irregular HRs were seen during prescan ECG monitoring, scans were performed in the axial mode (absolute delay acquisition for targeting end systole). For each patient, scan delay times between the start of contrast agent injection and the initiation of scanning were determined by the timing bolus technique. Briefly, after a bolus injection of 10 mL iopamidol (Pamiray, 370 mg iodine/mL; Dongkook Pharma, Seoul, Korea) followed by 20 mL of saline at 5 mL/s, optimal delay times were determined by automatic evaluation of the contrast enhancement in the ascending aorta. All CCTA scans were performed using the triple-phase injection method (70 mL of iopamidol followed by 30 mL of 30% blended iopamidol with saline and 20 mL of saline at 5 mL/s). Scan length usually included only the heart.

The APSCM was conducted with the CARE kV and simultaneous application of the CAREDose4D (Siemens Healthcare, Forchheim, Germany) [12]. The tube current was set to a reference of 250 mAs, and the tube potential was set to a reference of 120 kV. The CARE kV type setting was maintained on the “angiography” position for all patients. For the BMI-based group, kV and mAs were designated according to a previous BMI-based protocol [6]. Because all patients had a BMI higher than 30 kg/m^2^, the tube voltage was fixed at 120 kV for the BMI-based group. The tube current-time product was designated according to BMI (380 mAs for patients with a BMI ≤ 33 kg/m^2^ and 450 mAs for patients with a BMI > 33 kg/m^2^). Image reconstruction was performed from the raw datasets in the APSCM and BMI-based groups with a medium kernel (b36f) using filtered back projection. The reconstruction slice thickness was set to 0.75 mm with an increment of 0.5 mm, and the field of view included only the heart. Images were transferred to an offline workstation (Aquarius iNtuition, Ver 4.4.11, TeraRecon, San Mateo, CA, USA). Coronary arteries were evaluated with multiplanar reformat (MPR) and curved MPR images.

### Assessment of image quality

To acquire objective image quality parameters, the image noise, mean CT number, signal-to-noise ratio (SNR), and CNR were determined for all CCTA scans in the two groups. The image noise was defined as the standard deviation (SD) of a region of interest (ROI) drawn in the ascending aorta placed immediately cranial to the left coronary ostium. The ROI was drawn to be as large as possible while carefully avoiding inclusion of the aortic wall [13]. The CT numbers of the proximal right coronary artery (RCA) and left main coronary artery (LMA) were obtained by choosing the largest ROI possible without including the coronary vessel wall. The SNR was defined as the mean CT number of the proximal RCA and LMA, divided by image noise. The contrast enhancement of coronary arteries was defined as the difference between the CT numbers of the proximal RCA and LMA, with an ROI drawn in the perivascular fat tissue immediately adjacent to each coronary artery lumen [13]. The CNRs of the proximal RCA and LMA were calculated by dividing contrast enhancement values by image noise.

For subjective analysis of image quality, two observers who were blinded to patient demographics and CT parameters independently reviewed all CCTA scans. The images of each group were presented in random order. Image quality was subjectively rated for each of the four major coronary arteries using a four-point grading system, according to previously described methods [14, 15]. The overall image quality score was defined as the mean score of the four major coronary arteries (LMA, left anterior descending, left circumflex, and RCA). The lowest image quality score was defined as the lowest score of the four main coronary arteries. Interobserver variability was assessed for all patients.

### Estimation of radiation dose

The parameters collected for radiation dose analysis were the volume CT dose index (CTDI_vol_) and the dose-length product (DLP), both of which were obtained from the dose report on the CT scanner after each CCTA study. The effective dose (mSv) was calculated by multiplying the DLP by a cardiac conversion factor of 0.014 [16]. The size-specific dose estimate (SSDE) was also calculated based on the individual effective patient diameter, as measured from the scout images [17]. Only the radiation dose from the CCTA was assessed. Radiation doses associated with the localizer, timing bolus scan, or coronary calcium scan were not included.

### Statistical analysis

Since patients were not randomized to APSCM versus the BMI-based protocol for CCTA, we employed a propensity score matching method to compare the image quality and radiation doses of the two groups. The propensity score was defined as the conditional probability of undergoing CCTA with APSCM, given a vector of measured covariates. A multivariable logistic regression model was used to estimate the propensity score by using the baseline characteristics of age, sex, BMI, HR, and scan mode as covariates in the model. We matched the patients in the two groups based on their propensity score and compared image quality and radiation doses within each pair. To assess the degree of imbalance, we calculated the standardized mean difference (SMD) between the two groups before and after matching. The small (< 0.2) absolute values of SMD for each covariate indicate that the two matched groups were well balanced. We used R statistical software (version 3.2.2; R Foundation, Vienna, Austria) for propensity score matching. All other statistical analyses were performed using MedCalc, version 16.1.2 (MedCalc Software, Mariakerke, Belgium). Normally distributed data were identified using the Shapiro-Wilk W test. Continuous variables were presented as means ± SD or medians with interquartile ranges and were compared using the independent *t*-test or the Mann-Whitney U test, according to normality. When comparing two matched groups, we used the paired *t*-test for normally distributed data, and we used the Wilcoxon signed rank test for nonnormally distributed data. We evaluated statistical significance using χ^2^ statistics for categorical variables. Interobserver agreements on subjective image quality were quantified using linear weighted k statistics. To identify the predictors for 140 kV selection, we performed univariate and multiple logistic regression analyses. We omitted variables showing collinearity and entered only variables that were significant on univariate analysis into the multiple linear regression analysis. *P* values < 0.05 were considered statistically significant.

## Results

### Patient characteristics and CCTA parameters

The patient characteristics and CCTA parameters in the BMI-based group and the APSCM group are summarized in Table 1. No significant differences were observed between the two groups with respect to age, sex, BMI, or HR before or after matching. Scan modes were evenly distributed in both groups. In the BMI-based group, 120 kV was used in all 107 patients. However, low tube potentials (i.e., 80 or 100 kV) were automatically selected in 30 of the 96 patients (31.3%) in the APSCM group, whereas 120 kV and 140 kV were selected in 46 and 20 of the 96 patients (47.9% and 20.8%), respectively. The tube current-time product (mAs) was significantly lower in the APSCM group than in the BMI-based group, even after matching (*p* < .0001).

**Table 1:** Patient characteristics and coronary CT angiography parameters.

		*Before matching*			*After matching*		
	APSCM group (n = 96)	BMI-based group (n = 107)	SMD	*p* value	BMI-based group (n = 96)	SMD	*p* value
**Age, years**^†^	58.1 ± 13.8	56.4 ± 12.3	0.1315	0.354	57.0 ± 12.0	0.0734	0.53
**Male sex, n (%)**	47 (49.0)	47 (43.9)	0.0754	0.564	45 (46.9)	0.0415	0.87
**BMI (kg/m**^**2**^**)**	31.8 (30.8–33.2)	31.3 (30.8–32.9)	0.0278	0.695	31.3 (30.8–32.8)	0.0219	0.89
30–35 kg/m^2^	85 (88.5)	93 (86.9)		0.87	84 (87.5)		0.983
35–40 kg/m^2^	10 (10.4)	12 (11.2)			10 (10.4)		
>40 kg/m^2^	1 (1.0)	1 (0.9)			2 (2.1)		
**HR, beats/min**^‡^	60 (55–67.5)	60 (56–65)	0.1019	0.672	60 (56–65)	0.1009	0.35
**Scan mode**				0.915			> .9999
Flash mode, n (%)	29 (30.2)	32 (29.9)			30 (31.2)		
Axial mode, n (%)	67 (69.8)	75 (70.1)	0.0087		66 (68.8)	0.0226	
**Tube potential**				< 0.0001			< 0.0001
80 kV, n (%)	2 (2.1)	0 (0)			0 (0)		
100 kV, n (%)	28 (29.2)	0 (0)			0 (0)		
120 kV, n (%)	46 (47.9)	107 (100)			96 (100)		
140 kV, n (%)	20 (20.8)	0 (0)			0 (0)		
**Tube current-time product, mAs**^†^	363.0 ± 37.4	384.7 ± 30.6		< 0.0001	385.2 ± 31.7		< 0.0001

APSCM, automatic tube potential selection with tube current modulation; BMI, body mass index; CT, computed tomography; SMD, standardized mean difference, HR, heart rate.

† Data are expressed as means **±** standard deviations.

‡ Data are expressed as medians, with interquartile ranges in parentheses.

### Comparison of image quality and radiation dose in the overall study group

Quantitative measurements of image quality—including image noise, mean CT number, contrast enhancement of the coronary arteries, SNR, and CNR—were not significantly different between the BMI-based group and the APSCM group, either before or after matching (Table 2). There were no significant differences in subjective image quality between the two groups.

**Table 2:** Comparison of image quality between the BMI-based group and the APSCM group.

		*Before matching*		*After matching*	
	APSCM group (n = 96)	BMI-based group (n = 107)	*p* value	BMI-based group (n = 96)	*p* value
**Objective image quality**					
Image noise, HU	27.6 (24.7–37.2)	30.7 (27.6–35.7)	0.0521	30.4 (27.7–35.4)	0.63
CT number of coronary arteries, HU	352.8 (269.0–412.4)	330.6 (292.4–388.0)	0.5690	330.3 (293.5–382.3)	0.32
Contrast enhancement of coronary arteries, HU	408.9 (350.1–485.3)	404.2 (364.1–454.0)	0.7089	404.9 (367.6–446.4)	0.27
SNR	12.0 (9.5–13.9)	11.2 (9.1–12.9)	0.2107	11.2 (9.1–13.0)	0.4
CNR	13.8 (12.0–16.3)	13.3 (11.2–15.4)	0.1411	13.4 (11.3–15.4)	0.37
**Subjective Image Quality**					
Overall image quality score	3.5 (3.3–3.8)	3.5 (3.3–3.8)	0.8683	3.5 (3.3–3.8)	0.46
Lowest image quality score	3.0 (3.0–3.0)	3.0 (3.0–3.0)	0.8290	3 (3.0–3.0)	0.54

APSCM, automatic tube potential selection with tube current modulation; BMI, body mass index; CT, computed tomography; SNR, signal-to-noise ratio; CNR, contrast-to-noise ratio.

Data are expressed as medians, with interquartile ranges in parentheses.

Before matching, the radiation dose parameters were not significantly different between the two groups, with the exception of scan length (Table 3). The scan length was significantly different between the two groups, but this difference was not clinically significant. Among the patients examined in FLASH mode, the mean CTDI_vol_ and DLP values were significantly higher in the APSCM group than in the BMI-based group. However, after propensity score matching, including scan mode as a covariate, the radiation dose parameters were not significantly different between the two groups.

**Table 3:** Comparison of radiation exposure between the BMI-based group and the APSCM group.

		*Before matching*		*After matching*	
	APSCM group (n = 96)	BMI-based group (n = 107)	*p* value	BMI-based group (n = 96)	*p* value
**CTDI**_**vol**_**, mGy**					
All modes	18.4 (7.93–32)	27.3 (6.14–30.5)	0.978	26.9 (6.1–30.2)	0.792
Flash	6.72 (5.63–10.02)	6.12 (5.65–6.13)	0.04		
Axial	28.7 (18.1–38.8)	29.5 (26.5–31.6)	0.36		
**Scan length, cm**					
All modes	13.8 (13.8–17.5)	13.8 (12.5–17)	0.041	13.8 (12.9–17.1)	0.328
**Flash**	18.3 (17.1–19.7)	17.6 (16.1–18.2)	0.023		
Axial	13.8 (13.8–13.8)	13.8 (10.4–13.8)	0.017		
**DLP, mGy. cm**					
All modes	253.5 (143–435)	316 (115.3–408.8)	0.669	310.5 (112.0–407.3)	0.638
**Flash**	126 (108–174.3)	103 (91–111)	0.003		
Axial	397.5 (247–492.3)	378 (310.3–430.3)	0.806		
**Effective dose, mSv**					
All modes	3.5 (2.0–6.1)	4.4 (1.6–5.7)	0.669	4.3 (1.6–5.7)	0.638
**Flash**	1.8 (1.5–2.4)	1.4 (1.3–1.6)	0.003		
Axial	5.6 (3.5–6.9)	5.3 (4.3–6.0)	0.806		
**SSDE, mGy**					
All modes	21.1 (9.6–34.4)	30 (7.3–34.6)	0.788	29.7 (7.3–34.0)	0.987
Flash	7.62 (6.3–10.0)	6.59 (6.25–7.09)	0.059		
Axial	31.4 (19.4–39.2)	32.4 (29.5–36.2)	0.215		

APSCM, automatic tube potential selection with tube current modulation; BMI, body mass index; CTDI_vol_, volume CT dose index; DLP, dose-length product; SSDE, size-specific dose estimation.

Data are expressed as medians, with interquartile ranges in parentheses.

### APSCM subgroup analysis by tube potential

**Fig 2:**
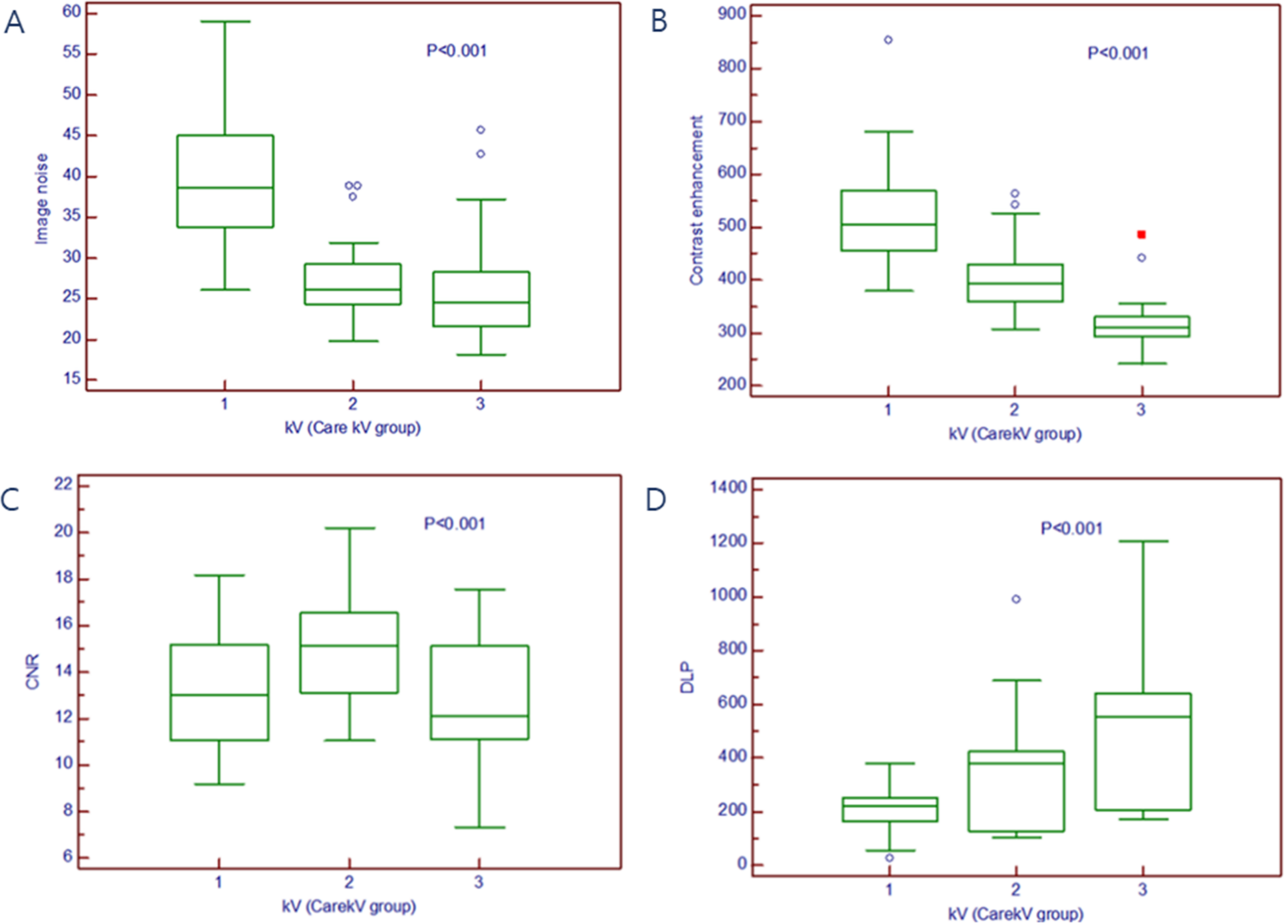
Image quality and radiation dose according to tube potential. (A) Image noise. (B) Contrast enhancement. (C) CNR. (D) DLP. Boxes represent the means and the first to third quartiles, mid-lines represent the medians, and whiskers indicate the minimum and maximum values. kV 1: 80 or 100 kV in the APSCM group; kV 2: 120 kV in the APSCM group; kV 3: 140 kV in the APSCM group. kV, kilovolt; APSCM, automatic tube potential selection with tube current modulation; BMI, body mass index (calculated as weight in kg/height in m^2^); CNR, contrast-to-noise ratio; SNR, signal-to-noise ratio; DLP, dose-length product.

When comparing objective image quality of different tube potential subgroups within the APSCM group, patients treated with higher tube potentials tended to show significantly lower image noise but significantly poorer contrast enhancement of coronary arteries compared with patients treated with lower tube potentials. Moreover, patients treated with a 140 kV tube potential showed significantly lower CNRs than patients treated with lower tube potentials (Fig 2, *p* < 0.001). In contrast, DLP increased as the tube potential increased (*p* < 0.001).

### Tube potential subgroup analysis after matching

**Fig 3:**
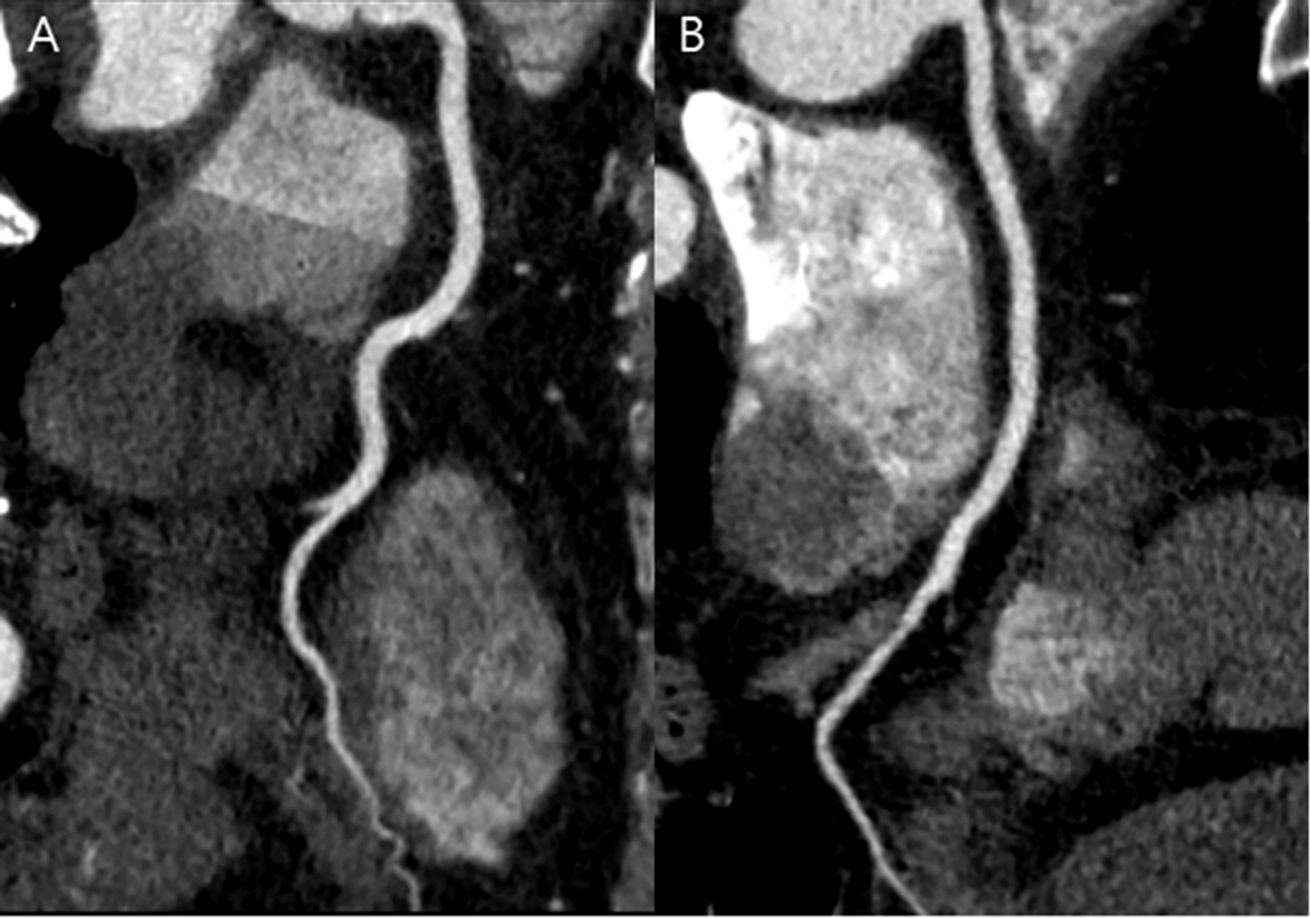
CT images of right coronary artery obtained in APSCM group with 100 kV and in matched BMI group. (A) Curved multiplanar reformatted image of RCA in a 43-year-old male in the APSCM group with a BMI of 30.1 kg/m^2^ and heart rate of 46 beats per minute. Image was obtained in the axial mode (100 kV and 350 mAs). Mean image quality score was 4, mean contrast enhancement was 580.3 HU, mean CT number was 483.9 HU, mean image noise was 40 HU. Mean CNR and SNR were 14.5 and 12.1, respectively. CTDI_vol,_ DLP, and effective dose were measured at 18.3 mGy, 253 mGy⋅cm, and 3.5 mSv, respectively. (B) Curved reformatted image of RCA in a 50-year-old male in the matched BMI group with a BMI of 31.6 kg/m^2^ and heart rate of 59 beats per minute. Image was obtained in the axial mode (120 kV and 380 mAs). Mean image quality score was 4, mean contrast enhancement was 397.1 HU, mean CT number was 324.4 HU, mean image noise was 29.1 HU. Mean CNR and SNR were 13.6 and 11.1, respectively. CTDI_vol,_ DLP, and effective dose were measured at 27.8 mGy, 383 mGy⋅cm, and 5.4 mSv, respectively. APSCM, automatic tube potential selection with tube current modulation; BMI, body mass index; CNR, contrast-to-noise ratio; CT, computed tomography; CTDI_vol_, volume CT dose index; DLP, dose-length product; RCA, right coronary artery; SNR, signal-to-noise ratio.

**Fig 4:**
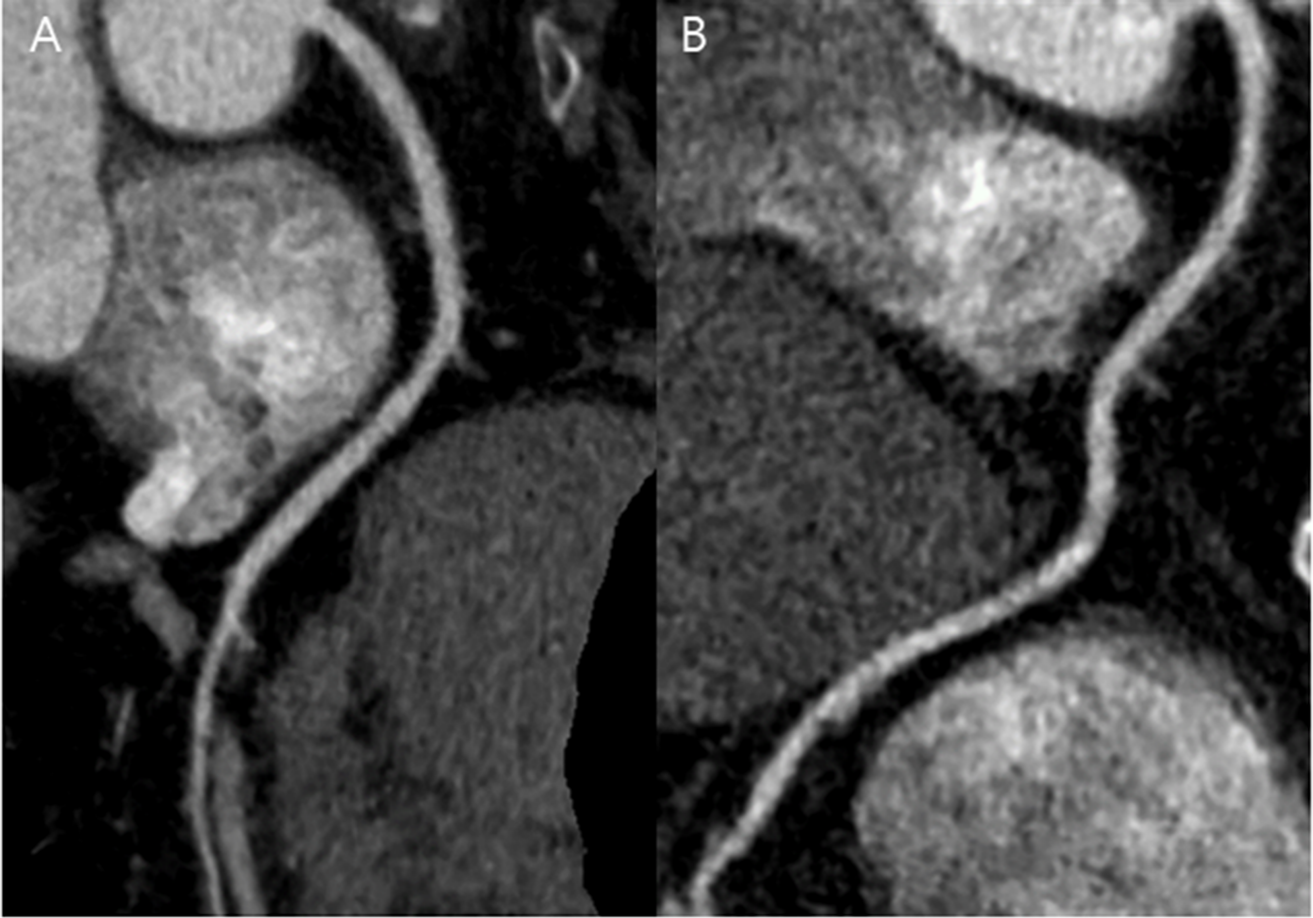
CT images in APSCM group with 120 kV and in matched BMI group. (A) Curved multiplanar reformatted image of RCA in a 64-year-old female in the APSCM group with a BMI of 31.5 kg/m^2^ and heart rate of 67 beats per minute. Image was obtained in the axial mode (120 kV and 348 mAs). Mean image quality score was 3.5, mean contrast enhancement was 412.2 HU, mean CT number was 313.1 HU, mean image noise was 25.3 HU. Mean CNR and SNR were 16.3 and 12.3, respectively. CTDI_vol_, DLP, and effective dose were measured at 27.0 mGy, 372 mGy⋅cm, and 5.2 mSv, respectively. (B) Curved multiplanar reformatted image of RCA in a 64-year-old female in the matched BMI group with a BMI of 30.8 kg/m^2^ and heart rate of 68 beats per minute. Image was obtained in the axial mode (120 kV and 380 mAs). Mean image quality score was 3.75, mean contrast enhancement was 433.7 HU, mean CT number was 347.7 HU, mean image noise was 39.6 HU. Mean CNR and SNR were 11.0 and 8.8, respectively. CTDI_vol_, DLP, and effective dose were measured at 26.1 mGy, 366 mGy⋅cm, and 5.1 mSv, respectively. APSCM, automatic tube potential selection with tube current modulation; BMI, body mass index; CNR, contrast-to-noise ratio; CT, computed tomography; CTDI_vol_, volume CT dose index; DLP, dose-length product; RCA, right coronary artery; SNR, signal-to-noise ratio.

**Fig 5:**
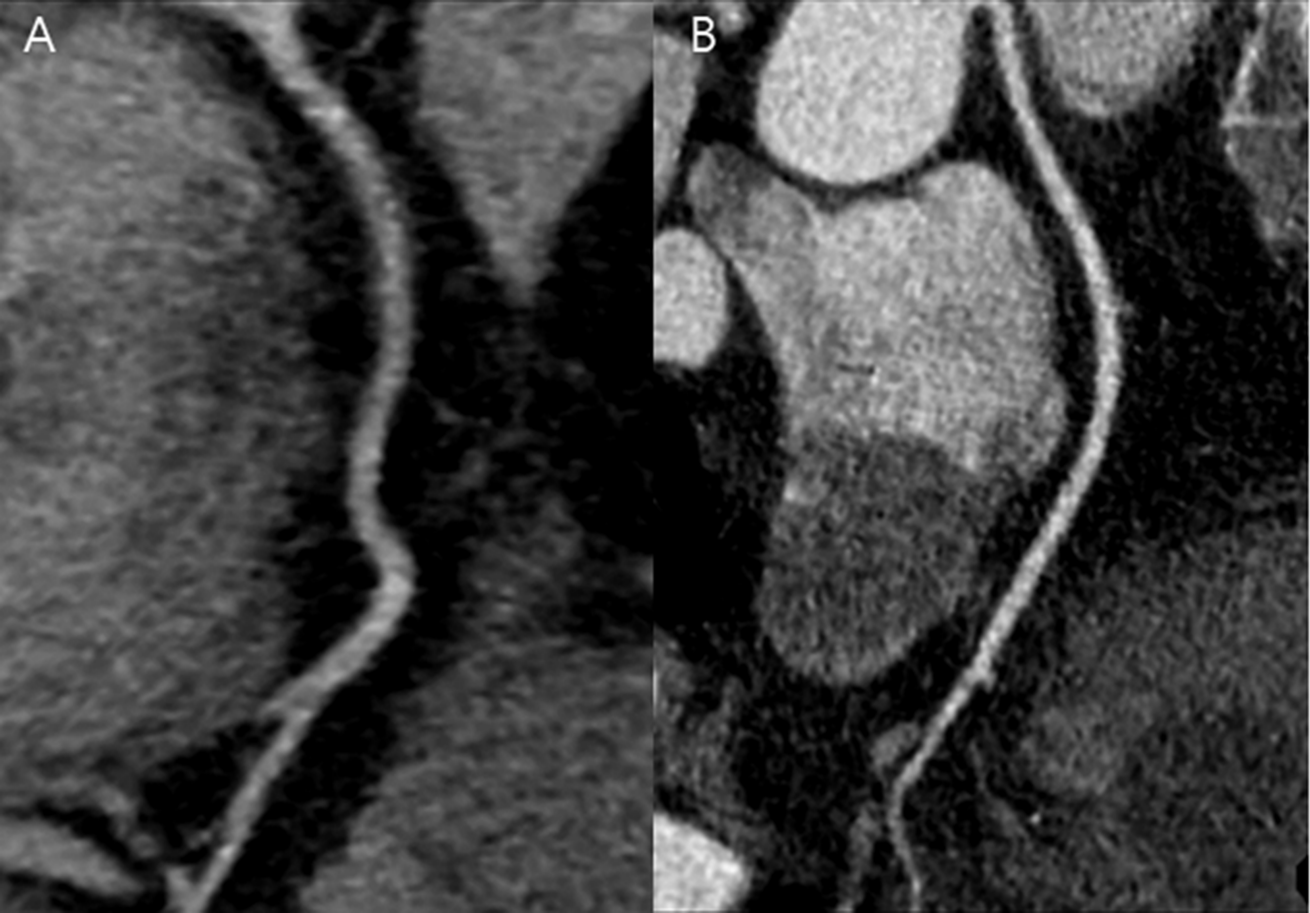
CT images in APSCM group with 140 kV and in matched BMI group. (A) Curved multiplanar reformatted image of RCA in a 56-year-old female in the APSCM group with BMI of 33.2 kg/m^2^ and heart rate of 73 beats per minute. Image was obtained in the axial mode (140 kV and 326 mAs). Mean image quality score was 3.25, mean contrast enhancement was 313.1 HU, mean CT number was 232.7 HU, mean image noise was 24.2 HU. Mean CNR and SNR were 12.9 and 9.6, respectively. CTDI_vol_, DLP, and effective dose were measured 35.3 mGy, 488 mGy⋅cm, and 6.8 mSv, respectively. (B) Curved multiplanar reformatted image of RCA in a 55-year-old female in the matched BMI group with a BMI of 33.9 kg/m^2^ and heart rate of 52 beats per minute. Image was obtained in the axial mode (120 kV and 450 mAs). Mean image quality score was 3.75, mean contrast enhancement was 411.0 HU, mean CT number was 322.8 HU, mean image noise was 40.4 HU. Mean CNR and SNR were 10.2 and 8.0, respectively. CTDI_vol_, DLP, and effective dose were measured 7.3 mGy, 135 mGy⋅cm, and 1.89 mSv, respectively. APSCM, automatic tube potential selection with tube current modulation; BMI, body mass index; CNR, contrast-to-noise ratio; CT, computed tomography; CTDI_vol_, volume CT dose index; DLP, dose-length product; RCA, right coronary artery; SNR, signal-to-noise ratio.

When comparing image quality and radiation dose between the APSCM subgroups and the matched BMI-based group, patients treated with 80 or 100 kV in the APSCM group exhibited significantly higher image noise, CT number, and contrast enhancement of coronary arteries (Table 4, *p* < 0.05; Fig 3) than matched patients in the BMI-based group. However, they also exhibited significantly lower CTDI_vol_, DLP, and SSDE values, thereby preserving the CNRs. Patients with 120 kV had significantly higher CNRs (*p* < 0.05) than matched patients in the BMI-based group treated with a comparable radiation dose (Fig 4). Patients with 140 kV showed significantly lower image noise, but significantly higher CTDI_vol_, DLP, and SSDE than patients in the matched BMI-based group (*p* < 0.05, Fig 5), without significant CNR improvement. There was no significant difference in subjective image quality score between the APSCM group and the matched BMI-based group, regardless of tube potential.

**Table 4:** Subgroup analysis of image quality and radiation dose according to tube potential.

	*80 or 100 kV (n = 30)*	*Matched BMI (n = 30)*	p *value*	*120 kV (n = 46)*	*Matched BMI (n = 46)*	p *value*	*140 kV (n = 20)*	*Matched BMI (n = 20)*	p *value*
**Objective Image Quality**									
Image noise, HU	38.6 (33.8–45.0)	30.4 (27.7–35.4)	0.0002	26.1 (24.3–29.2)	31.9 (28.5–35.7)	0.0001	24.5 (21.6–28.3)	29.2 (27.5–36.5)	0.0441
CT number of coronary arteries, HU	438.3 (383.7–488.0)	330.3 (293.5–382.2)	<0.0001	331.5 (291.0–375.7)	330.2 (291.9–396.6)	0.8698	240.1 (227.5–262.8)	324.0 (285.2–394.3)	0.0006
Contrast enhancement of coronary arteries, HU	505.4 (455.2–569.7)	404.9 (367.6–446.4)	<0.0001	692.7 (357.0–430.0)	399.1 (365.5–443.9)	0.6700	309.9 (293.4–330.5)	381.8 (347.7–479.8)	0.0009
SNR	11.0 (9.8–13.2)	11.2 (9.1–13)	0.7971	12.5 (10.3–14.3)	11.1 (8.8–12.3)	0.0175	9.4 (8.4–11.5)	10.8 (9.4–14.0)	0.2455
CNR	13.0 (11.1–15.2)	13.4 (11.3–15.4)	0.5304	15.1 (13.1–16.2)	13.1 (11.0–15.2)	0.0060	12.1 (11.1–15.1)	12.9 (11.4–16.3)	0.5958
**Subjective Image Quality**									
Overall image quality score	3.5 (3.3–3.8)	3.5 (3.3–3.8)	0.5531	3.5 (3.3–3.8)	3.5 (3.25–3.75)	0.2672	3.0 (3.0–3.0)	3.3 (2.8–3.6)	0.3591
Lowest image quality score	3.0 (3.0–3.0)	3.0 (3.0–3.0)	0.8040	3.0 (3.0–3.0)	3.0 (3.0–3.0)	0.6051	3.0 (3.0–3.0)	3.0 (2.0–3.0)	0.8311
**Radiation dose**									
CTDI_vol_, mGy	16.4 (14.0–18.3)	26.7 (6.1–29.7)	0.0285	28.0 (7.0–31.5)	27.2 (14.0–31.6)	0.9869	40.8 (10.1–43.2)	26.6 (6.1–30.1)	0.0484
Scan length, cm	13.8 (13.8–14.3)	14.2 (13.8–17.2)	0.5440	13.8 (13.8–17.9)	13.8 (10.4–16.5)	0.0180	13.8 (13.8–17.6)	13.8 (13.8–17.0)	0.5459
DLP, mGy.cm	219.0 (163.0–253.0)	298.0 (105.0–409.0)	0.0175	381.0 (128.0–424.0)	299.5 (145.0–411.0)	0.8441	552.5 (207.5–640.5)	322.5 (109.0–385.5)	0.0094
Effective dose, mSv	3.1(2.3–3.5)	4.2 (1.5–2.9)	0.0175	5.3 (1.8–5.9)	4.2 (2.0–5.8)	0.8441	7.7 (2.9–9.0)	4.5 (1.5–5.4)	0.0094
SSDE, mGy	18.4 (14.8–20.6)	30.0 (6.6–34.7)	0.0243	31.1 (7.7–34)	29.5 (16.2–36.1)	0.8741	42.4 (11.2–46.3)	30.3 (7.4–32.1)	0.0484

BMI, body mass index (calculated as weight in kg/height in m^2^); CNR, contrast-to-noise ratio; CT, computed tomography; CTDI_vol_, volume CT dose index; DLP, dose-length product; SNR, signal-to-noise ratio; SSDE, size-specific dose estimation.

Data are expressed as medians, with interquartile ranges in parentheses.

### Predictors for selection of 140 kV

Patients with a high body weight or a large body size (weight > 90 kg, BMI > 32.9 kg/m^2^, or effective diameter > 32.2 cm) had increased odd ratios for selection of 140 kV in the APSCM group (Table 5). Multivariate logistic regression analysis revealed that BMI > 32.9 kg/m^2^ and effective diameter > 32.2 cm were independent predictors for 140 kV selection.

**Table 5:** Predictors for selection of 140 kV.

	Univariate OR (95% CI)	*p* value	Multivariate OR (95% CI)	*p* value
Male sex	1.36 (0.05–3.65)	0.544	N/A	N/A
Weight > 90 kg	4.23 (1.46–12.29)	0.008	N/A	N/A
BMI > 32.9 kg/m^2^	9 (3.01–26.9)	<0.001	6.6 (2.12–20.53)	0.0011
Effective diameter > 32.2 cm	13.1 (1.66–102.9)	0.015	8.42 (1.02–69.74)	0.0483
AP diameter	3.91 (1.3–11.9)	0.016	N/A	N/A
Lateral diameter	3.93 (1.41–11)	0.009	N/A	N/A

AP, antero-posterior; BMI, body mass index; CI, confidence interval; OR, odds ratio.

## Discussion

Our study shows that the use of APSCM for CCTA in obese patients does not result in a significant difference in image quality or radiation dose compared with the use of a BMI-based protocol. The effects of APSCM on image quality and radiation dose differed according to the selected tube potential. While comparable image quality can be maintained, a 39% decrease in SSDE was observed when 80 or 100 kV was selected. When 120 kV was selected, partial improvement of objective image quality was observed without any significant increase in the radiation dose. However, when 140 kV was selected, the SSDE increased by up to 40% without any obvious improvement in image quality. Moreover, 140 kV tended to be selected in patients who had a high BMI and in patients with a large body size (BMI > 32.9 kg/m^2^, effective diameter > 32.2 cm).

The results of our study contrast with those of previous studies demonstrating that the use of APSCM in CCTA significantly improved image quality and/or reduced the radiation dose. This discrepancy may be due to differences in the patient characteristics. In the previous studies, the mean BMI of the study population ranged from 24.2 to 29.9 kg/m^2^, and 140 kV was selected in 0 to 7.9% of the study population [5, 9, 11, 18–20]. In contrast, only obese patients (BMI > 30 kg/m^2^, mean = 32.2 kg/m^2^) were enrolled in our study; thus, 140 kV was selected more frequently (20.8%) than in previous studies. Comparing the 140 kV group with the matched BMI controls, the SSDE was significantly increased by 40% without any significant benefit in image quality.

The APSCM automatically selects the most appropriate tube potential and the mAs setting according to the patient’s body habitus (attenuation and size estimated from the scout image) and the exam purpose such that a user-chosen CNR is maintained. Although only obese patients (BMI greater than 30 kg/m^2^) were included in this study, distribution of the selected tube potentials was relatively even among the patients. These data indicate that BMI is not an ideal measurement for accurately representing a patient’s body habitus because multiple other anthropometric factors such as body size and body attenuation can affect the image quality of CCTA scans [21]. That is, patients with similar BMIs can be assigned to different tube potentials. In our study, patients with an effective diameter larger than 32.9 cm were more likely to undergo CCTA with 140 kV as determined by APSCM.

Previous studies have observed that the quality of CCTA images is reduced when patients are overweight or obese. In particular, image quality parameters such as image noise, CNR, and SNR all worsen as BMI increases [22–24]. To compensate for the degradation of image quality caused by high BMI, several protocols using increased voltage (120 kV or 140 kV) have been proposed for obese patients [25–27]. However, Lee et al. found that CCTA using 140 kV yielded no significant beneficial effect on image quality compared with CCTA at 120 kV, despite inflicting a 35.3% increase in effective radiation dose. Lee et al. also found that CCTA at 120 kV had a higher iodine contrast attenuation, which was sufficient to compensate for the increased image noise, thus resulting in similar CNRs and SNRs compared with those obtained by CCTA at 140 kV [28].

Considering the results of previous studies [5, 8–11, 19, 20], it may be reasonable to conclude that APSCM significantly reduces radiation exposure while preserving image quality of CCTA in the general population, largely due to the selection of low kV settings. However, the findings presented here indicate that more frequent selection of 140 kV leads to unfavorable effects on patients who have a high BMI and a large body size. In one large observational study, the effect of APSCM on radiation dose was shown to vary with body region and type of CT examination [20]. Specifically, in most body regions and for most types of CT examination, the CTDI_vol_ was decreased due to more frequent use of 100 kV. However, increased radiation doses were observed for renal stone protocols, and for thoracic and lumbar spine examination. These increased doses were accompanied by an increased frequency of 140 kV selection by APSCM; the precise reasons underlying these increases are not yet known [20]. Based on these results, more thoughtful consideration is needed to determine the appropriate indications and applications of APSCM.

This study does have some limitations. First, it was a retrospective study, and data were obtained from a single institution. However, to avoid selection bias, patients were consecutively enrolled during a specific time period; moreover, all images were analyzed in a blinded fashion, i.e., without information regarding the patient group. Second, because only a limited number of patients (n = 27; 19 patients in the BMI-based group and 8 patients in the APSCM group) underwent invasive coronary angiography, the diagnostic accuracies of the two groups were not compared. However, our study focused only on the effects of APSCM on image quality and radiation dose; to determine the effect of APSCM on diagnostic accuracy, future large-scale investigations are required. Third, we could not use other strategies for radiation dose reduction, such as iterative reconstruction techniques, because these techniques were not available during the period in which patients in the BMI-based group were examined. Many studies have demonstrated the effects of iterative reconstruction in decreasing image noise and the potential to reduce radiation dose while maintaining image quality. A recent meta-analysis demonstrated that a 48% radiation dose reduction can be achieved with the use of iterative reconstruction compared with filtered back projection [29]. We suggest that combined use of APSCM and iterative reconstruction would achieve further dose reduction with acceptable image quality or improved image quality with a similar radiation dose for obese patients, and we expect future studies to resolve this issue. Finally, although the reduced dose or contrast agent can be achieved by use of low kV, we did not investigate the feasibility of using a low-dose contrast agent with simultaneous use of APSCM [30].

In conclusion, the use of APSCM for CCTA in obese patients did not significantly reduce the radiation dose or improve image quality compared with matched obese patients in the BMI-based group. Our data also indicate that it is better to avoid the application of APSCM when 140 kV is automatically selected because this value inflicts an increased radiation dose without any significant improvement in image quality.

## Supporting information

S1 File Attached file contains data of the image quality and radiation dose of CCTA from obese patients with BMI-based protocol (group 1) and those with APSCM (group 2). (XLSX)
